# Oxalic acid–pyridine-4-carbonitrile (1/2)

**DOI:** 10.1107/S1600536812019137

**Published:** 2012-05-05

**Authors:** Wen-Ni Zheng

**Affiliations:** a College of Chemistry and Chemical Engineering, Southeast University, Nanjing 210096, People’s Republic of China

## Abstract

In the title compound, 2C_6_H_4_N_2_·C_2_H_2_O_4_, the oxalic acid mol­ecule lies about an inversion center. The pyridine ring of the pyridine-4-carbonitrile mol­ecule is almost planar, the largest deviation from the least-squares plane being 0.006 (1) Å; the nitrile N atom deviates from this plane by 0.114 (1) Å. In the crystal, the oxalic acid mol­ecules and the pyridine-4-carbonitrile mol­ecules form stacks. Neighboring mol­ecules within the stacks are related by translation in the *a* direction, with inter­planar distances of 3.183 (1) and 3.331 (2) Å, respectively. Each oxalic acid mol­ecule forms strong O—H⋯N hydrogen bonds with two mol­ecules of pyridine-4-carbonitrile. Besides this, there are also weak C—H⋯O inter­actions.

## Related literature

For the structures and ferroelectric properties of related compounds, see: Fu *et al.* (2011*a* ▶,*b* ▶,*c* ▶); Dai & Chen (2011 ▶); Xu *et al.* (2011 ▶); Zheng (2011 ▶). For standard bond lengths, see: Allen *et al.* (1987 ▶).
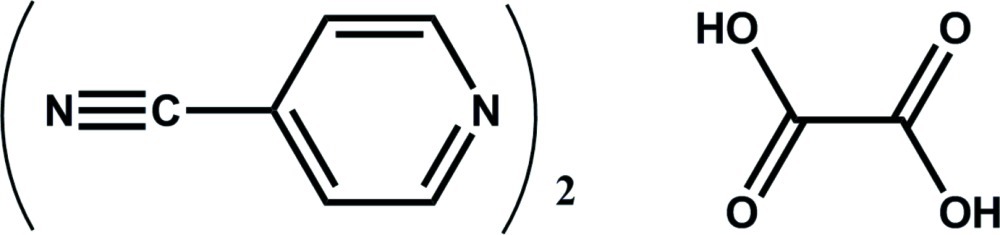


## Experimental

### 

#### Crystal data

C_6_H_4_N_2_·0.5C_2_H_2_O_4_
*M* *_r_* = 149.13
Triclinic, 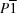

*a* = 3.6842 (6) Å
*b* = 7.5816 (5) Å
*c* = 12.4511 (1) Å
α = 78.258 (1)°
β = 85.301 (1)°
γ = 82.547 (1)°
*V* = 337.08 (6) Å^3^
*Z* = 2
Mo *K*α radiation
μ = 0.11 mm^−1^
*T* = 298 K
0.10 × 0.03 × 0.03 mm

#### Data collection

Rigaku SCXmini Mercury2 diffractometer
Absorption correction: multi-scan (*CrystalClear*; Rigaku, 2005 ▶) *T*_min_ = 0.910, *T*_max_ = 1.000
3634 measured reflections
1528 independent reflections
1268 reflections with *I* > 2σ(*I*)
*R*_int_ = 0.024

#### Refinement

*R*[*F* ^2^ > 2σ(*F* ^2^)] = 0.036
*wR*(*F* ^2^) = 0.101
*S* = 1.04
1528 reflections
103 parameters
1 restraint
H atoms treated by a mixture of independent and constrained refinement
Δρ_max_ = 0.46 e Å^−3^
Δρ_min_ = −0.27 e Å^−3^

### 

Data collection: *CrystalClear* (Rigaku, 2005 ▶); cell refinement: *CrystalClear*; data reduction: *CrystalClear*; program(s) used to solve structure: *SHELXS97* (Sheldrick, 2008 ▶); program(s) used to refine structure: *SHELXL97* (Sheldrick, 2008 ▶); molecular graphics: *SHELXTL* (Sheldrick, 2008 ▶); software used to prepare material for publication: *SHELXTL*.

## Supplementary Material

Crystal structure: contains datablock(s) I, global. DOI: 10.1107/S1600536812019137/yk2054sup1.cif

Structure factors: contains datablock(s) I. DOI: 10.1107/S1600536812019137/yk2054Isup2.hkl

Supplementary material file. DOI: 10.1107/S1600536812019137/yk2054Isup3.cml

Additional supplementary materials:  crystallographic information; 3D view; checkCIF report

## Figures and Tables

**Table 1:** Hydrogen-bond geometry (Å, °)

*D*—H⋯*A*	*D*—H	H⋯*A*	*D*⋯*A*	*D*—H⋯*A*
O1—H1⋯N1	0.82 (1)	1.80 (1)	2.6173 (12)	176 (2)
C4—H4*A*⋯O1^i^	0.93	2.48	3.3640 (13)	160

Symmetry code: (i) 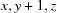.

**Fig. 1.**
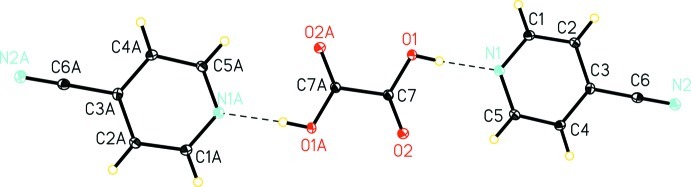
A view of the (2/1) molecular complex with the atomic numbering scheme. The displacement ellipsoids were drawn at the 30% probability level.

**Fig. 2.**
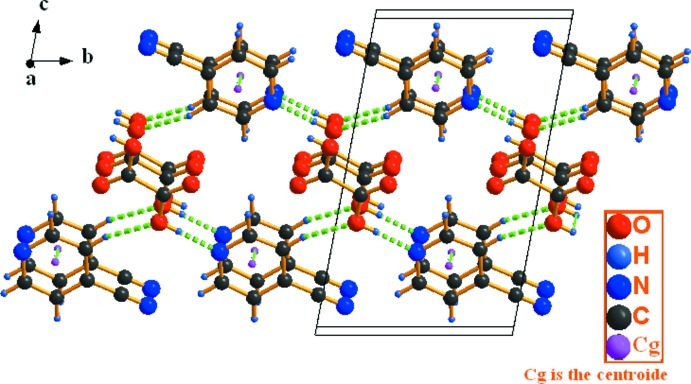
The crystal packing of the title compound viewed along the a axis and showing the O—H···N, π···π and C—H···O interactions.

## supplementary crystallographic information

### Comment

Simple organic salts containing strong intermolecular H-bonds have attracted an attention as materials which display ferroelectric-paraelectric phase transitions (Fu *et al.*, 2011*a*,*b*,*c*). With the purpose of obtaining crystals of organic salts exhibiting ferroelectric phase transitions, various organic compounds have been studied and the series of new materials have been elaborated (Dai & Chen, 2011; Xu *et al.*, 2011; Zheng, 2011). Herewith we present the synthesis and crystal structure of the title molecular complex, pyridine-4-carbonitrile–oxalic acid (2/1).

All bond lengths and angles in the studied structure have expected values (Allen *et al.*, 1987). The dihedral angle between the pyridine ring and the oxalic acid molecule is 10.29 (8)°. The H atoms of oxalic acid are involved in strong intramolecular O—H···N hydrogen bonds (Fig. 1 and Table 1), with the O···N distance of 2.617 (3)Å. The weak intermolecular C—H···O interaction is also presented in this structure, with C4···O1 = 3.364 (2)Å. The crystal packing is further stabilized by the π···π interactions between the pyridine rings of the neighbouring pyridine-4-carbonitrile molecules (Fig. 2).

### Experimental

The oxalic acid (10 mmol), pyridine-4-carbonitrile (20 mmol) and ethanol (50 mL) were put into a 100mL flask. The mixture was stirred at 60°C for 2 h, and then the precipitate was filtered off. Colourless crystals suitable for X-ray diffraction were obtained by slow evaporation of the solution.

### Refinement

All the H atoms attached to C atoms were placed into the idealized positions and treated as riding with C—H = 0.93 Å and with *U_iso_*(H)=1.2*U_eq_*(C). The positional parameters of the H atom attached to oxygen were refined freely, and at the last stage of the refinement, they were restrained with the H—O = 0.82 (2)Å and with *U_iso_*(H)=1.5*U_eq_*(O).

### Crystal data

**Table:**

C_6_H_4_N_2_·0.5C_2_H_2_O_4_	*Z* = 2
*M**_r_* = 149.13	*F*(000) = 154
Triclinic, *P*1	*D*_x_ = 1.469 Mg m^−^^3^
Hall symbol: -P 1	Mo *K*α radiation, λ = 0.71073 Å
*a* = 3.6842 (6) Å	Cell parameters from 1528 reflections
*b* = 7.5816 (5) Å	θ = 2.9–27.5°
*c* = 12.4511 (1) Å	µ = 0.11 mm^−^^1^
α = 78.258 (1)°	*T* = 298 K
β = 85.301 (1)°	Block, colourless
γ = 82.547 (1)°	0.10 × 0.03 × 0.03 mm
*V* = 337.08 (6) Å^3^	

### Data collection

**Table:**

Rigaku SCXmini Mercury2 diffractometer	1528 independent reflections
Radiation source: fine-focus sealed tube	1268 reflections with *I* > 2σ(*I*)
Graphite monochromator	*R*_int_ = 0.024
Detector resolution: 13.6612 pixels mm^-1^	θ_max_ = 27.5°, θ_min_ = 2.9°
ω scans CCD profile fitting	*h* = −4→4
Absorption correction: multi-scan (*CrystalClear*; Rigaku, 2005)	*k* = −9→9
*T*_min_ = 0.910, *T*_max_ = 1.000	*l* = −16→16
3634 measured reflections	

### Refinement

**Table:**

Refinement on *F*^2^	Primary atom site location: structure-invariant direct methods
Least-squares matrix: full	Secondary atom site location: difference Fourier map
*R*[*F*^2^ > 2σ(*F*^2^)] = 0.036	Hydrogen site location: inferred from neighbouring sites
*wR*(*F*^2^) = 0.101	H atoms treated by a mixture of independent and constrained refinement
*S* = 1.04	*w* = 1/[σ^2^(*F*_o_^2^) + (0.065*P*)^2^] where *P* = (*F*_o_^2^ + 2*F*_c_^2^)/3
1528 reflections	(Δ/σ)_max_ < 0.001
103 parameters	Δρ_max_ = 0.46 e Å^−^^3^
1 restraint	Δρ_min_ = −0.27 e Å^−^^3^

### Special details

**Table:**

Geometry. All s.u.'s (except the s.u. in the dihedral angle between two l.s. planes) are estimated using the full covariance matrix. The cell s.u.'s are taken into account individually in the estimation of s.u.'s in distances, angles and torsion angles; correlations between s.u.'s in cell parameters are only used when they are defined by crystal symmetry. An approximate (isotropic) treatment of cell s.u.'s is used for estimating s.u.'s involving l.s. planes.
Refinement. Refinement of F^2^ against ALL reflections. The weighted R-factor wR and goodness of fit S are based on F^2^, conventional R-factors R are based on F, with F set to zero for negative F^2^. The threshold expression of F^2^ > 2sigma(F^2^) is used only for calculating R-factors(gt) etc. and is not relevant to the choice of reflections for refinement. R-factors based on F^2^ are statistically about twice as large as those based on F, and R- factors based on ALL data will be even larger.

### Fractional atomic coordinates and isotropic or equivalent isotropic displacement parameters (Å^2^)

**Table:**

	*x*	*y*	*z*	*U*_iso_*/*U*_eq_	
O1	0.8019 (2)	0.10266 (10)	0.37597 (6)	0.0195 (2)	
H1	0.753 (4)	0.2095 (6)	0.3465 (11)	0.029*	
O2	1.0250 (2)	0.22804 (10)	0.50208 (6)	0.0196 (2)	
N1	0.6137 (3)	0.43796 (12)	0.27800 (8)	0.0157 (2)	
N2	0.1419 (3)	1.12502 (13)	0.09513 (8)	0.0207 (2)	
C4	0.5310 (3)	0.75440 (15)	0.28546 (9)	0.0147 (3)	
H4A	0.5545	0.8461	0.3231	0.018*	
C1	0.4759 (3)	0.47781 (15)	0.17793 (9)	0.0158 (3)	
H1A	0.4592	0.3835	0.1417	0.019*	
C3	0.3848 (3)	0.79346 (14)	0.18234 (9)	0.0141 (3)	
C5	0.6404 (3)	0.57355 (15)	0.33014 (9)	0.0152 (3)	
H5A	0.7362	0.5455	0.3992	0.018*	
C2	0.3578 (3)	0.65365 (14)	0.12654 (9)	0.0150 (3)	
H2A	0.2638	0.6777	0.0573	0.018*	
C6	0.2532 (3)	0.97886 (14)	0.13355 (9)	0.0161 (3)	
C7	0.9557 (3)	0.09891 (14)	0.46785 (8)	0.0140 (3)	

### Atomic displacement parameters (Å^2^)

**Table:**

	*U*^11^	*U*^22^	*U*^33^	*U*^12^	*U*^13^	*U*^23^
O1	0.0299 (5)	0.0114 (4)	0.0174 (4)	−0.0004 (3)	−0.0102 (4)	−0.0010 (3)
O2	0.0269 (5)	0.0140 (4)	0.0189 (4)	−0.0026 (3)	−0.0066 (3)	−0.0033 (3)
N1	0.0164 (5)	0.0147 (5)	0.0158 (5)	−0.0021 (4)	−0.0022 (4)	−0.0020 (4)
N2	0.0244 (6)	0.0171 (5)	0.0210 (5)	−0.0016 (4)	−0.0067 (4)	−0.0029 (4)
C4	0.0142 (5)	0.0151 (5)	0.0159 (5)	−0.0027 (4)	−0.0002 (4)	−0.0049 (4)
C1	0.0169 (6)	0.0153 (5)	0.0164 (5)	−0.0030 (4)	−0.0017 (4)	−0.0048 (4)
C3	0.0118 (5)	0.0130 (5)	0.0167 (5)	−0.0018 (4)	−0.0004 (4)	−0.0011 (4)
C5	0.0157 (5)	0.0169 (6)	0.0131 (5)	−0.0027 (4)	−0.0025 (4)	−0.0019 (4)
C2	0.0156 (6)	0.0169 (6)	0.0126 (5)	−0.0025 (4)	−0.0023 (4)	−0.0024 (4)
C6	0.0162 (6)	0.0177 (6)	0.0155 (5)	−0.0043 (4)	−0.0014 (4)	−0.0042 (4)
C7	0.0144 (5)	0.0143 (5)	0.0131 (5)	−0.0010 (4)	−0.0012 (4)	−0.0024 (4)

### Geometric parameters (Å, º)

**Table:**

O1—C7	1.3113 (12)	C4—H4A	0.9300
O1—H1	0.822 (2)	C1—C2	1.3871 (15)
O2—C7	1.2075 (13)	C1—H1A	0.9300
N1—C5	1.3403 (14)	C3—C2	1.3981 (15)
N1—C1	1.3462 (13)	C3—C6	1.4505 (14)
N2—C6	1.1503 (14)	C5—H5A	0.9300
C4—C5	1.3910 (15)	C2—H2A	0.9300
C4—C3	1.3940 (14)	C7—C7^i^	1.557 (2)
			
C7—O1—H1	107.8 (11)	N1—C5—C4	122.85 (10)
C5—N1—C1	118.83 (9)	N1—C5—H5A	118.6
C5—C4—C3	117.70 (10)	C4—C5—H5A	118.6
C5—C4—H4A	121.1	C1—C2—C3	117.75 (10)
C3—C4—H4A	121.1	C1—C2—H2A	121.1
N1—C1—C2	122.74 (10)	C3—C2—H2A	121.1
N1—C1—H1A	118.6	N2—C6—C3	178.67 (12)
C2—C1—H1A	118.6	O2—C7—O1	126.63 (10)
C4—C3—C2	120.13 (10)	O2—C7—C7^i^	121.93 (12)
C4—C3—C6	120.12 (9)	O1—C7—C7^i^	111.44 (11)
C2—C3—C6	119.74 (10)		

Symmetry code: (i) −*x*+2, −*y*, −*z*+1.

### Hydrogen-bond geometry (Å, º)

**Table:**

*D*—H···*A*	*D*—H	H···*A*	*D*···*A*	*D*—H···*A*
O1—H1···N1	0.82 (1)	1.80 (1)	2.6173 (12)	176 (2)
C4—H4*A*···O1^ii^	0.93	2.48	3.3640 (13)	160

Symmetry code: (ii) *x*, *y*+1, *z*.
